# Multi‐Omics Analysis of Gut Microbiome and Host Metabolism in Different Populations of Chinese Alligators (*alligator sinensis*) During Various Reintroduction Phases

**DOI:** 10.1002/ece3.71221

**Published:** 2025-04-09

**Authors:** Chong Wang, Changcheng Li, Fuyong You, Yongkang Zhou, Genjun Tu, Ruoya Liu, Pingsi Yi, Xiaobing Wu, Haitao Nie

**Affiliations:** ^1^ The Anhui Provincial Key Laboratory of Biodiversity Conservation and Ecological Security in the Yangtze River Basin College of Life Sciences, Anhui Normal University Wuhu China; ^2^ Anhui Chinese Alligator National Nature Reserve Xuancheng Anhui China

## Abstract

Reintroduction plays a significant role in the self‐maintenance and reconstruction of wild animal populations, serving as a communication bridge between captive and wild animals. The Chinese alligator ( *Alligator sinensis* ) is a distinct and endangered reptile species found in China. The mechanisms by which artificially bred Chinese alligators adapt following their release into the wild remain poorly understood. This study aims to elucidate the alterations in gut microbiomes and metabolic phenotypes of Chinese alligators during their reintroduction. During the Chinese alligator's reintroduction, Fusobacterium and *Cetobacterium* became more abundant, while typical pathogens declined significantly. The gut type of the Chinese alligator changed from *Acinetobacter* to *Cetobacterium*. The construction of the gut microbial community was dominated by neutral (random) processes and shifted towards deterministic processes with the progression of reintroduction. In terms of species function, reintroduction significantly upregulated the expression of host immune‐related genes and significantly decreased the expression of gut bacterial pathogenic genes and antibiotic resistance genes. Metagenomic and metabolomic KEGG enrichment analyses indicate that glucoside hydrolase families 13 and 23—alongside glycolysis and gluconeogenesis pathways—may play pivotal roles in energy metabolism, host‐pathogen interactions, and homeostasis maintenance for Chinese alligators. Differential metabolite analysis identified significant upregulation of metabolites related to neuroendocrine immune modulation and significant down‐regulation of anti‐inflammatory metabolites during Chinese alligator reintroduction. Association analysis showed that there were significant co‐metabolic effects between microorganisms and metabolites, which coordinated host adaptive interaction. This study provides insights into the synergistic mechanisms of host adaptation and wild environment adaptation for Chinese alligators.

## Graphical abstract

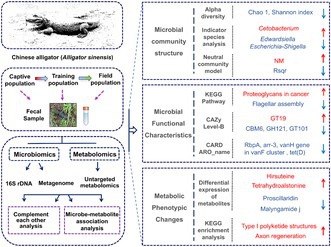

## 1 Introduction

Captive breeding and reintroduction play crucial roles in the conservation and management of wildlife species. The reintroduction of captive‐bred individuals into their natural habitats can effectively restore degraded populations and reverse the trend of biodiversity loss (Huang et al. 2021). In this framework, captive populations act as a genetic reservoir for wild populations, preserving critical genetic diversity to support population recovery through future reintroductions. Nevertheless, captivity can profoundly alter the host‐associated microbiota through various mechanisms, including changes in food sources, exposure to environmental microbial banks, co‐housing with other species, and the administration of antibiotics (Clayton et al. 2016; Chong et al. 2019; Trevelline et al. 2019). The gut microbial community has an important impact on host function, and its changes may lead to increased potential dysbiosis and susceptibility to gut‐related diseases (e. g., obesity, inflammatory bowel disease, autoimmune disease) (Schmidt et al. 2019; Round and Mazmanian 2009; Moloney et al. 2014; Thaiss et al. 2016).

Current studies suggest that gut microbiota may mediate adaptive phenotypic plasticity in response to environmental changes (Alberdi et al. 2016). In vertebrate models, cold exposure experiments in mice ( *Mus musculus* ) have demonstrated that microbial community restructuring regulates energy metabolism through white adipose tissue browning (Chevalier et al. 2015), while cross‐species comparative studies further reveal differential modulation of microbiome structure by host phylogeny and diet (Youngblut et al. 2019). In invertebrate systems, the case of the wood‐feeding beetle ( *Odontotaenius disjunctus* ) illustrates the anatomical basis of host‐microbial functional synergy through compartmentalized gut structures enabling lignocellulose decomposition (Ceja‐Navarro et al. 2019). Notably, these studies collectively highlight two evolutionarily conserved mechanisms: (1) microbiome‐mediated phenotypic plasticity via metabolic intermediates, as exemplified by the microbiome‐gut‐brain axis (Davidson et al. 2020); and (2) co‐adaptation between host anatomical features and microbial ecological niches, consistent with Shapira (2016) multilayered selection model. These mechanistic frameworks provide critical insights for investigating microbial regulation of physiological adaptation across diverse taxa. These considerations are particularly important for reintroduced species, as they may be influenced by various factors, including environmental changes during the conservation process, which can reduce the likelihood of successful reintroduction (Tang et al. 2023; Jiménez and Sommer 2017). As a key indicator for the reintroduction of captive animals, the gut microbiota provides effective monitoring targets and intervention strategies (Huang et al. 2024). Monitoring gut microbiota diversity and keystone taxa can reveal adaptation challenges in reintroduced animals, as dysbiosis correlates with environmental stress and reduced fitness (West et al. 2019). Targeted interventions—including dietary prebiotics to enrich beneficial taxa, probiotics, and fecal microbiota transplantation to restore dysbiotic communities—collectively improve host health and ecological resilience (West et al. 2019; McKenzie et al. 2018).

Gut microbiota dynamics are critical for evaluating wildlife adaptation. Studies in crocodilians reveal captivity‐induced microbiome shifts linked to health risks (Willson et al. 2019) and gut bacteria producing bioactive metabolites that may enhance environmental stress tolerance (Khan et al. 2021). Yet such insights remain unexplored in endangered species with active reintroduction efforts. The Chinese alligator ( *Alligator sinensis* ) is a unique and endangered reptile in China, due to habitat loss, fragmentation, and human activities. The International Union for Conservation of Nature (IUCN) Red List categorizes the Chinese alligator as ‘Critically Endangered (CR)’ (Jiang and Wu 2018). As the flagship species for protecting the Yangtze River wetland ecosystem in China, the Chinese alligator holds significant ecological conservation value. Captive‐bred individuals can serve as genetic resources for the field population, which facilitates the effective preservation of their genetic diversity and supports the recovery of the field population. Over recent years, as the population has gradually increased, some of the released groups have adapted to the field environment and begun to reproduce and lay eggs. The large‐scale release of the Chinese alligator has positively impacted the long‐term evolutionary dynamics and developmental potential of the population. However, there is currently a lack of comprehensive performance evaluation and monitoring protocols post‐release, and the extent of adaptation and response mechanisms of both the Chinese alligators and their gut microbes to the environment remains unclear.

The gut microbiome and host metabolism exhibit dynamic interactions during environmental adaptation, with microbial compositional shifts potentially driving metabolic reprogramming that impacts host phenotypes (Liu et al. 2023; Visconti et al. 2019). Through the in‐depth integration of stool‐based multi‐omics approaches, it is possible to comprehensively characterize the interactions between hosts and their gut microbiomes (Sauceda et al. 2022).

In this study, fresh fecal samples from Chinese alligators at different release stages—captivity, rewilding training, and field population—were subjected to a combined microbiome and metabolomics analysis. The objectives were as follows: (1) To elucidate the systematic interplay and regulatory dynamics between gut microbiota, function, and metabolites in Chinese alligators across various release stages; (2) To investigate the classification, functional diversity, and the dynamics of complexity within the gut microbiota; (3) To gain an in‐depth understanding of the potential etiologies, environmental adaptation capabilities, and metabolic profiles of Chinese alligator populations. This study aims to elucidate the determinants of gut microbial community structure and function, thereby establishing a foundation for the synergistic integration of breeding research with field conservation efforts. The goal is to develop more scientific and effective conservation strategies aimed at maintaining and reconstructing self‐sustaining populations of field Chinese alligators.

## 2 Materials and Methods

### 2.1 Subject and Sample Collection

Taking into account the variations in the primary food types and environmental conditions at different stages of the Chinese alligator's release (captive, rewilding training, and field release), we classified individuals into three groups under distinct management protocols: the Captive Group (artificially maintained in concrete ponds with daily standardized feeding and temperature‐controlled hibernation), the Rewilding‐training Group (transitioned to semi‐natural ponds with biweekly live prey supplementation and ambient thermal conditions), and the Field Release Group (established in restored wetlands through food chain reconstruction, relying on natural prey availability with minimal human intervention). In May 2024, we collected 39 fresh fecal samples (13 per group) from these groups at the Anhui Chinese Alligator National Nature Reserve, with outdoor sampling conducted using liquid nitrogen tanks followed by laboratory storage at −80°C.

Following the quality assessment of the fecal samples, 16S rRNA sequencing was conducted to characterize the gut microbial community structure. Utilizing the sequencing data, we employed the supervised method Distict within the MicroPITA software to select samples for further analysis. MicroPITA analyses were necessary to optimize resource allocation while maintaining statistical power. Specifically, the supervised District algorithm in MicroPITA was employed to prioritize samples that maximally represented inter‐group variance (Tickle et al. 2013). As a result, 12 samples (*N* = 4 × 3) and 18 samples (*N* = 6 × 3) were chosen for metagenomic and untargeted metabolic sequencing analyses to elucidate the functional and metabolic characteristics of the Chinese alligator's gut microbiome. Complete screening metrics parameters are provided in Table S1.

### 2.2 DNA Extraction, Sequencing, Assembly, and Annotation

#### 2.2.1 16 s rDNA Sequencing and Extraction

DNA was extracted using the HiPure Fecal DNA Extraction Kit (Model D3241, Guangzhou Meji Biotechnology Co. Ltd., China). Each stool sample was homogenized and extracted to yield 150–200 mg for subsequent analysis. The quality of the extracted DNA was assessed using a NanoDrop microspectrophotometer (Model NanoDrop 2000, Thermo Fisher Scientific) and agarose gel electrophoresis (Model DYY‐6C, Beijing Liuyi Instrument Factory) to ensure it met the experimental requirements. PCR amplification was performed using a PCR instrument (Model ETC811, Beijing Dongsheng Xingye Scientific Instrument Co. Ltd.) and quantified with a Qubit 3.0 fluorometer (Thermo Fisher Scientific). In terms of reagent selection, PCR‐related reagents were sourced from New England Biolabs (USA), and AMPure XP magnetic beads, utilized in the recovery and purification process, were provided by Beckman Coulter Company. The V3–V4 region was targeted for sequencing, and the 16S rDNA was amplified using specific primers with unique barcodes. The forward primer is 341F‐ ‘CCTACGGGNGGCWGCAG’ and the reverse primer is 806R‐ ‘GGACTACHVGGGTATCTAAT’ with a primer length of about 466 base pairs. Subsequently, amplification was performed following a 50 μL PCR reaction system and corresponding PCR procedures were set. After completing the PCR amplification, the second round of amplification products were purified using AMPure XP Beads. Subsequently, quantification was performed using ABI StepOnePlus Real‐Time PCR System (Life Technologies, USA) to ensure library quality. Finally, pooling on‐machine sequencing was performed according to the PE250 mode of Novaseq6000 to obtain high‐quality sequencing data.

#### 2.2.2 Metagenomic Sequencing and Extraction

Library Quantification and Sequencing: The primary instruments utilized were the Agilent 2100 Bioanalyzer (Agilent Technologies, USA) and the ABI StepOnePlus Real‐Time PCR System (Life Technologies, USA). The main reagents used were the DNA 1000 assay kit (Cat. No. 5067‐1504) and the High Sensitivity DNA assay kit (Cat. No. 5067–4626), both from Agilent Technologies, USA. DNA 1000 assay kit The detectable sample fragment size range is 25–1000 bp and the concentration range is 0.1–50 ng/μL; High Sensitivity DNA assay kit is the accurate quantification of pg samples, and the detectable sample fragment size range is 50–7000 bp and the concentration range is 5–500 pg/μL. Finally, the libraries were quantified using the ABI StepOnePlus Real‐Time PCR System (Life Technologies, USA). Following this, the quantified libraries were pooled in PE150 mode for sequencing on the NovaSeq X Plus.

#### 2.2.3 Non‐Targeted Metabolomics

Before injecting samples into the LC–MS/MS (liquid chromatography–tandem mass spectrometry), quality control (QC) samples were added. Approximately 100 mg of each sample was analyzed using an Agilent 1290 Infinity LC (UHPLC) HILIC column with gradient elution. The samples were kept in a 4°C autosampler during analysis. Samples were analyzed randomly to minimize the impact of instrumental signal fluctuation. QC samples were interspersed within the sample cohort to monitor the system's stability and the reliability of the experimental data. The raw data were converted to MzML format using ProteoWizard (v3.0.6428), and peak alignment, retention time correction, and peak area extraction were performed using the XCMS program (v3.7.1). The data processed by XCMS underwent metabolite structure identification, data preprocessing (including null filtering, filling, and data filtering), and, finally, data analysis.

### 2.3 Analytic Procedure

#### 2.3.1 16 s rRNA for Data Analysis

Data quality control was performed using Usearch software (v11.0.667), which included clustering and deconvolution to obtain representative sequences and abundance information for each OTU (operational taxonomic unit). Representative OTU sequences were aligned to the SILVA database (v138.1), and species classification was annotated using the RDP annotating software (v2.2) with a confidence threshold of 0.8. Dilution curves were plotted with the ggplot2 package (v2.2.1) in R to assess sequencing depth and diversity. The Chao1 and Shannon diversity indices were calculated using QIIME 2 (v2020.2), and the Alpha diversity indices were compared with the Vegan R package (v2.5.3). QIIME 2 (v2023.9) was also used to display species classification and abundance. Statistical analysis of significant differences between groups was performed with one‐way ANOVA, and the Tukey's HSD test was used to assess differences in means when significant differences were detected (*p* < 0.05). For beta diversity analysis, based on OTU and species abundance tables, NMDS analysis was conducted using the R Vegan package (v2.5.3) and plotted with ggplot2 (v2.2.1). The Bray‐Curtis distance and Anosim test (non‐parametric analysis) were performed with the Vegan package (v2.5.3). The UpSet plot package (v1.6.16) in R was used to analyze shared and unique OTUs between groups. The abundance differences of species across multiple groups were analyzed using the Kruskal–Wallis H test with the R vegan package (v2.5.3). Gut analysis involved using the clusterSim package (v0.51.5) to calculate the Calinski‐Harabasz index to determine the optimal number of clusters, and the Jensen‐Shannon distance was calculated using the ade4 package (1.7.22) for cluster analysis. Neutral community model analysis used beta distributions to fit the relationship between species occurrence frequency and mean relative abundance. Model fit was performed using the minpack.lm package (v1.2.4) in R to compare predicted and actual frequencies of occurrence and calculate the model's goodness of fit. The grid package (v4.4.1) was used to plot the occurrence frequency against mean relative abundance and to illustrate the model predictions' curves and confidence intervals.

#### 2.3.2 Metagenomics and Analysis

The raw Illumina sequencing data were filtered using FASTP (v0.18.0), and the clean reads were assembled using MEGAHIT (v1.1.2) to obtain long contiguous sequences, which were termed contigs. Gene prediction was performed on these contigs using MetaGeneMark (v3.38). Sequences of at least 300 bp in length were selected, merged into clusters using CD‐HIT (v4.6), and the longest sequence from each cluster was chosen as the Unigene. A set of non‐redundant gene sequences was obtained through realignment of reads, statistics, and filtering with Bowtie (v2.2.5). DIAMOND (v0.9.24) was employed to annotate the Unigenes against the KEGG (Kyoto Encyclopedia of Genes and Genomes), CAZy (Carbohydrate‐Active enZYmes Database), and CARD (Comprehensive Antibiotic Research Database) databases. Based on gene abundance, the functional abundance of each category was calculated. Circos (v0.69–3) was used to visualize the functional abundance in a circos map. Additionally, the R package vegan (v2.5.3) was utilized to perform the Kruskal–Wallis H test and analyze the abundance differences between species across multiple groups.

#### 2.3.3 Non‐Targeted Metabolomics Analysis

To maximize metabolite coverage, the analysis utilized both positive (positive ion mode, POS) and negative (negative ion mode, NEG) ionization modes. Quality control samples were processed using the R package gmodels (v2.18.1) for quality control (QC) and principal component analysis (PCA). Partial least squares‐discriminant analysis (PLS‐DA) was conducted with the R package ropls (v1.36.0), including cross‐validation and permutation testing to ensure the reliability of the PLS‐DA model. The variable importance in projection (VIP) values from the multivariate PLS‐DA and the *T*‐test *p*‐values from the univariate statistical analysis were combined to identify metabolites that were significantly different between comparison groups. A significant difference was determined using a threshold of a VIP value greater than 1 in the PLS‐DA model and a *T*‐test *p*‐value of less than 0.05. The abundance of metabolites found to be significantly different within the same group was normalized using z‐score normalization and plotted against the VIP values from the PLS‐DA analysis. Compound IDs (C_id) corresponding to the qualitative metabolite results were obtained from the KEGG database. After applying correction for multiple testing, pathways with a *Q*‐value of less than 0.05 were defined as significantly enriched in the differentially expressed metabolites.

#### 2.3.4 Association Analysis

The differential species and metabolites at the genus level, identified by a Kruskal–Wallis test with a significance threshold of *p* < 0.05 and a VIP value greater than 1, along with a *p*‐value less than 0.05, will be further analyzed using the Pearson correlation method. The calculations for these correlations will be performed using the corrplot package (v4.5.0). The correlations with a weight of Cor > 0.8 and a *p*‐value less than 0.01 will be considered strong and displayed using a weighted network graph, which will be plotted with the igraph package (v2.1.1).

## 3 Results

### 3.1 Characteristics of Gut Microbial Diversity and Composition Changes During the Reintroduction of Chinese Alligators

The amplification and sequencing of the V3‐V4 region of the 16S rRNA gene from fecal samples of different release stages (captive, rewilding training, and field release) of Chinese alligators were conducted to deeply analyze the characteristics of changes in gut microbial community structure. After quality filtering and analysis, a total of 4192 operational taxonomic units (OTUs) were obtained, with an average of 107 OTUs per sample. The Sobs dilution curves showed that these sample sizes provided sufficient sequence to penetrate the bacterial diversity (Figure S1a). Based on the results of taxonomic annotation, these OTUs were found to be affiliated with 25 phyla and 355 genera, respectively. The Upset plot shows 224 OTUs shared between the fecal samples from the three stages, while the captive, training, and field populations had 172,112, and 59 unique OTUs, respectively (Figure S1b).

**FIGURE 1:**
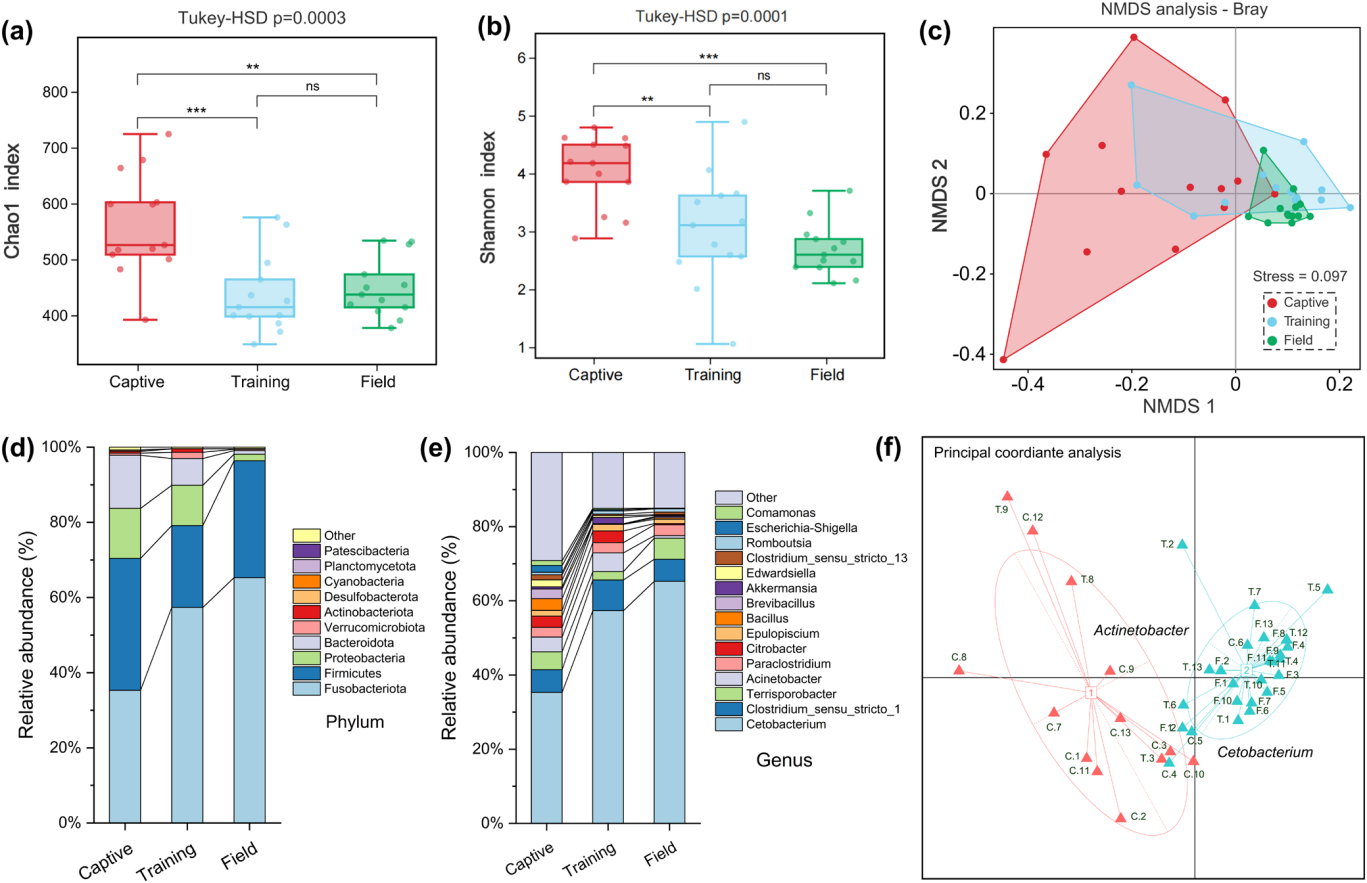
Analysis of α diversity indices based on Tukey HSD: (a) Chao 1 index; (b) Shannon index; (c) NMDS analysis based on Bray‐Curtis distance; (d) stacked bar chestart of species composition at the TOP10 phylum level; (e) stacked bar chart of species composition at the TOP15 genus level; (f) gut microbiota type analysis.

Based on Tukey's HSD test, Chao 1 and Shannon indices were selected to assess the trend of gut microbial community richness and diversity during the reintroduction of the Chinese alligator (Figure 1a,b). The study indicated that both the richness and diversity of the training and field populations were significantly lower than those of the captive population (*p* < 0.01), whereas no significant differences were observed between the domesticated and field populations. Significant differences in microbial community composition among the three groups were revealed by NMDS analysis (Figure 1c) and Anosim test (Figure S1c) with an R value of 0.268 and a *p*‐value of 0.001, indicating a reduction in the differences within microbial communities between groups. In the NMDS analysis (Stress = 0.097), it was found that the captive population and the field population were obviously divided into two groups, and the domesticated population played an obvious transition role between the two, and the distance was close to the field population, indicating that the species similarity was relatively high.

In terms of gut microbial composition (Figure 1d,e), at the phylum level in the Top 10 (Figure 1d), the relative abundance of Fusobacteria significantly increased (*p* < 0.01) in the gut microorganisms of captive individuals when they were released into the natural environment. This change resulted in a transition of the major groups in the gut microbiome from being predominantly composed of Fusobacteria and Bacteroidetes to being completely dominated by Fusobacteria. Among the three groups, the total relative abundance proportion of Fusobacteria, Firmicutes, Proteobacteria, and Bacteroidetes combined exceeded 97%, representing the typically dominant bacterial phyla. By the Kruskal–Wallis test, it was found that the four categories were significantly different between the three groups (*p* < 0.05). Additionally, significant changes in the Firmicutes to Bacteroidetes ratio (F/B ratio) were observed in the gut bacterial flora of Chinese alligators (Figure S1e). Specifically, the F/B ratios for captive Chinese alligators, training populations, and field populations were 9.40, 17.39, and 60.20, respectively. Further Tukey's HSD test revealed that there was no significant difference in the F/B ratio between captive and training populations, but significant differences were found between captive and field populations (*p* < 0.001) as well as between training and field populations (p < 0.01).

At the genus taxonomic level of Top15 (Figure 1e), the dominant bacteria (relative abundance > 5%) in the gut of the Chinese alligator included *Cetobacterium*, *Clostridium sensu stricto 1*, *Terrisporobacter*, and *Acinetobacter*. According to the Kruskal–Wallis test (Figure S1f), the relative abundance of *Cetobacterium* in the field population was found to increase significantly (*p* < 0.05). These significant changes in the relative abundance may be strongly correlated with the transition in environmental and feeding conditions. Meanwhile, we noticed that the relative abundance of some typical opportunistic pathogens, including *Edwardsiella*, *Escherichia–Shigella*, and *Comamonas*, significantly decreased (*p* < 0.05), indicating that the gut pathogenic risk in the Chinese alligator significantly decreased during training and field release.

**FIGURE 2:**
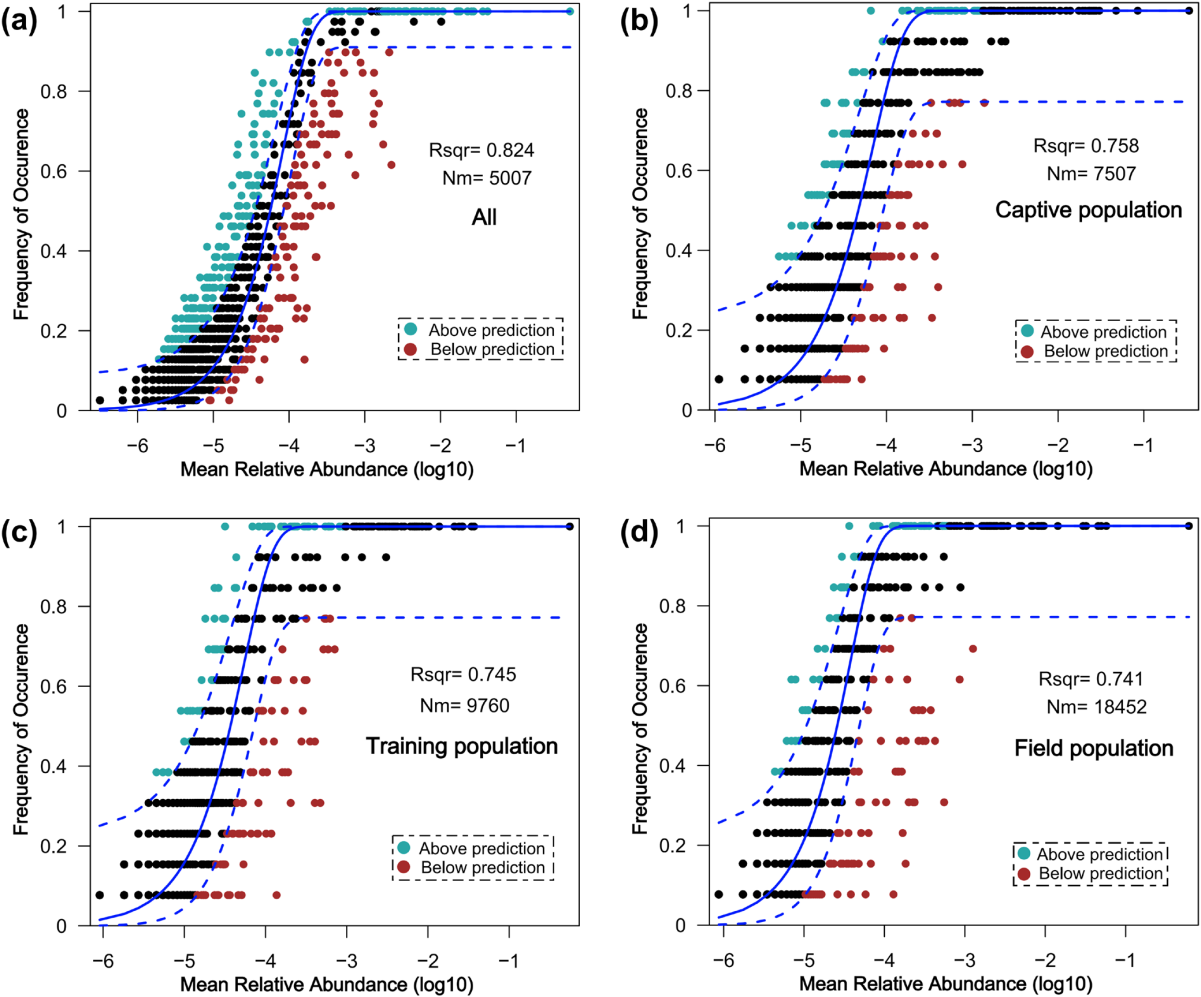
Neutral community model (NCM) showing the predicted occurrence frequency of gut microbial communities in all (a), captive (b), training (c), and field (d) populations of Chinese alligator. The blue solid line represents the best model fit, while the blue dashed lines indicate the 95% confidence intervals around the predictions. OTUs with occurrence frequencies higher or lower than the NCM predictions are shown in green and red, respectively.

In addition, samples were clustered based on the relative abundance composition at the genus level of the microbial community. The Jensen‐Shannon divergence (JSD) distance was calculated to perform a gut‐type analysis. First, the Calinski‐Harabasz (CH) index was determined from the clustering results, with the highest CH index corresponding to the optimal number of sample clusters, which was found to be 2. We clustered the samples using the Partitioning Around Medoids (PAM) algorithm and generated the PCoA map (Figures 1 and 2). The results revealed that the captive population was characterized by a high prevalence of *Acinetobacter*, while the field population predominantly contained *Cetobacterium*. In the domesticated population, a smaller portion was primarily associated with *Acinetobacter*, while the majority showed a distinct shift. This indicates that in the process of field release, the gut microbiota of the Chinese alligator underwent a gradual transition from a microbiome dominated by *Acinetobacter* to one where *Cetobacterium* became prevalent and subsequently diversified.

### 3.2 Fit the Neutral Model of Microbial Community Assembly in the Gut of the Chinese Alligator

Through the neutral community model (NCM) (Figure 2), we observed and analyzed the species abundance distribution of the Chinese alligator gut microbiome. In the figure, the Rsqr index reflects the overall goodness of fit for the neutral model. The NCM for integration, captive, training, and field populations had Rsqr values of 0.824, 0.758, 0.745, and 0.741, respectively. This suggests that the construction process of these microbial communities aligns with the neutral model, indicating that stochastic processes dominate community assembly. This emphasizes the potential importance of stochastic processes in the assembly of gut microbial communities. However, with the process of release in the field, the influence of stochastic processes on the microbial community formation decreased, while the influence of deterministic processes gradually increased, explaining the adaptation process of the Chinese alligator gut microbiota to environmental factors. In the neutral model, Nm quantifies estimates of community dispersal and establishes an association between frequency of occurrence and regional relative abundance. For the three populations described above, the values of Nm are 7507, 9760, and 18,452, respectively. These differences suggest that during the reintroduction process in the wild, the significant changes in environmental conditions lessen the restrictions on the dispersal and migration of the intestinal microbial species of the Chinese alligator, thereby leading to a greater intensification of structural differences between different communities.

### 3.3 Functional Divergence of Gut Microbial Communities During Reintroduction of Chinese Alligators

**FIGURE 3:**
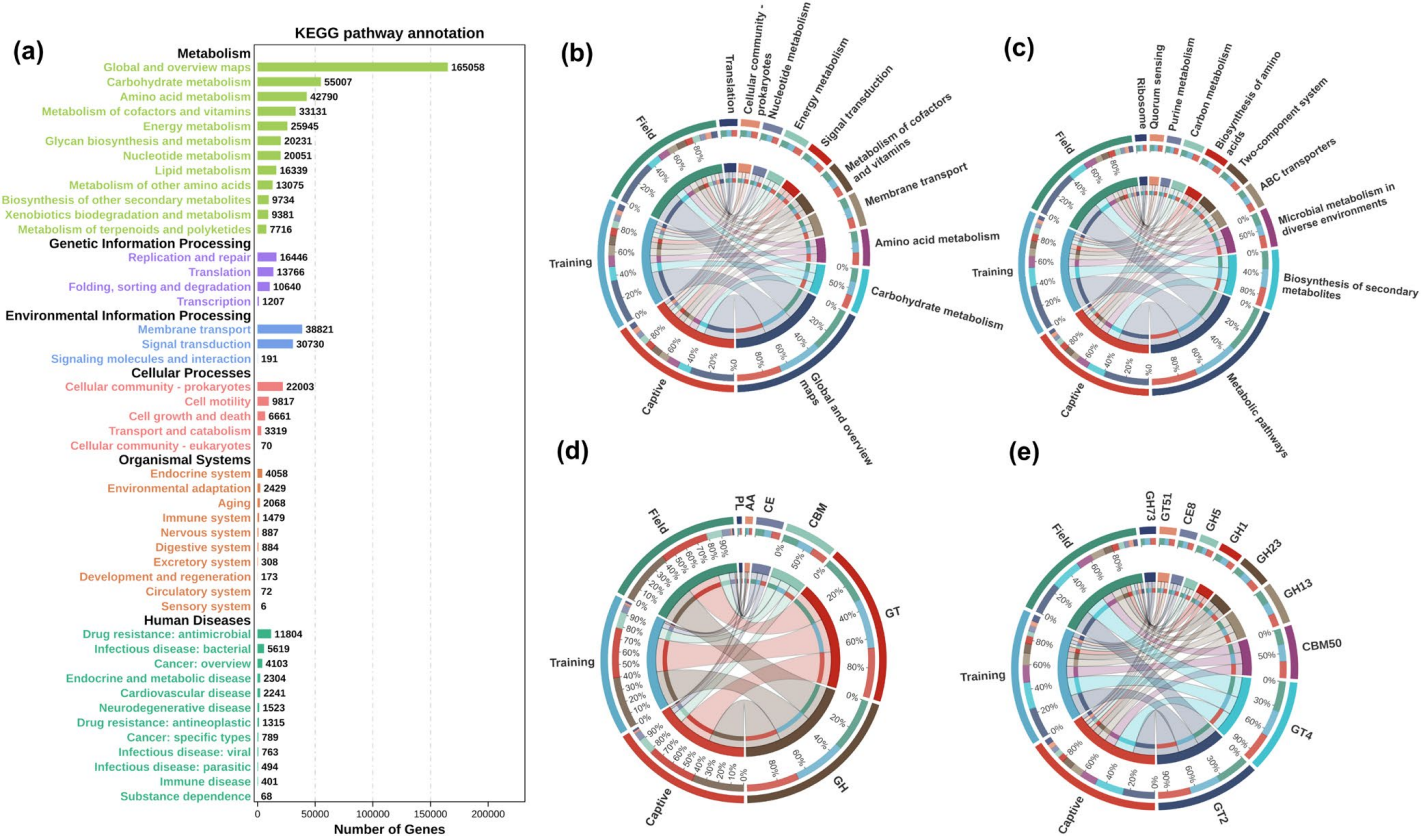
(a) Bar chart showing the number of annotated KEGG genes in all samples; (b, c) Circos diagrams displaying the distribution characteristics of the top 10 and top 15 enriched genes at level‐B (b) and pathway (c) classification levels; (d, e) functional distribution characteristics of the CAZy database: Circos diagrams showing level‐A (d) and level‐B (e).

The de novo assembly included 111,348 scaffolds assemblies with an average length of 1605.80 bp (Table S2). In the gene functional annotation, 86.12% of the genes were classified into the KEGG database, and 16.63% and 4.42% were classified into the CAZy and CARD databases, respectively (Table S3). First, the differentially expressed genes that were filtered were subjected to Kyoto Encyclopedia of Genes and Genomes (KEGG) gene function annotation analysis (Figure 3a). The annotation results were classified into six major categories, with the largest number of genes being enriched in the pathways of metabolism and environmental information processing. The enrichment differences in the Top 10 KEGG pathways at the Level‐B and Pathway classification levels for three groups were demonstrated by Circos plots (Figure 3b,c). At the secondary classification level of metabolism pathways, the highest enrichment of metabolic pathways was observed in the Global and overview maps and Carbohydrate metabolism. Among these, Amino acid metabolism decreased as the process of reintroduction progressed, while the number of genes involved in Signal transduction and Translation increased with the progression. At the Pathway classification level, the pathways with the highest enrichment of genes were Metabolic pathways and Biosynthesis of secondary metabolites. The number of genes involved in the Two‐component system decreased as the process of reintroduction progressed, while the number of genes involved in Quorum sensing and Ribosome increased with the progression of the process. The Upset plot demonstrated the differences in the number of enriched pathways at the Pathway classification level, revealing that the field population had significantly more pathways than the captive and domesticated populations (Figure S2a). Further analysis using the Kruskal–Wallis test at the Pathway classification level (Figure S2b) found that pathways such as Flagellar assembly and Biosynthesis of various other secondary metabolites were significantly downregulated with the progression of the reintroduction process (*p* < 0.05), whereas the pathway Proteoglycans in cancer was significantly upregulated.

Gene enrichment to the Carbohydrate‐Active enZyme (CAZy) database was performed, and annotations and statistical analyses were carried out at the Level‐A and Level‐B classification levels using Circos plots (3d, 3e). It was found that at the Level‐A classification level, the number of genes enriched in glycoside hydrolases (GH) was the highest, with GH13 and GH23 being the dominant CAZyme families of GH. At the Level‐B classification level, the families GT2 and GT4 of glycosyl transferases (GT) were dominant. Analysis of the gene enrichment differences at the Level‐B classification level using an UpSet plot revealed that the number of genes in the Chinese alligator's gut decreased as it transitioned from a captive state to domestication and wild environments (Figure S2c). Further analysis using the Kruskal–Wallis test at the Level‐B classification level (Figure S2d) found that GT19 was significantly upregulated (*p* < 0.05) with the process of reintroduction, while CBM6, GH121, and GT101 were significantly downregulated.

In addition, genes were annotated to the Comprehensive Antibiotic Resistance Database (CARD) to characterize resistance gene changes in the gut microbes of Chinese alligators. The UpSet plot found that ARO genes were downregulated in the gut from captivity to domestication and field environments (Figure S2e). The Circos plot displayed the functional distribution patterns of the Top 10 abundant antibiotic resistance genes (ARGs) (Figure S2f), with the expression of macB being the highest. Further analysis using the Kruskal–Wallis test (Figure S2g) revealed that 17 antibiotic resistance genes showed significant differences, most of which were upregulated in the domesticated population and then significantly downregulated in the field population. Among them, RbpA, arr‐3, the vanH gene in the vanF cluster, and tet(D) were significantly downregulated with the process of reintroduction. The relative abundance and trends of these enriched pathways, enzymes, and genes may have a significant impact on the physiological and ecological adaptation mechanisms of the Chinese alligator.

### 3.4 Characteristics of Host Metabolic Phenotype Changes During the Reintroduction Process of the Chinese Alligator

**FIGURE 4:**
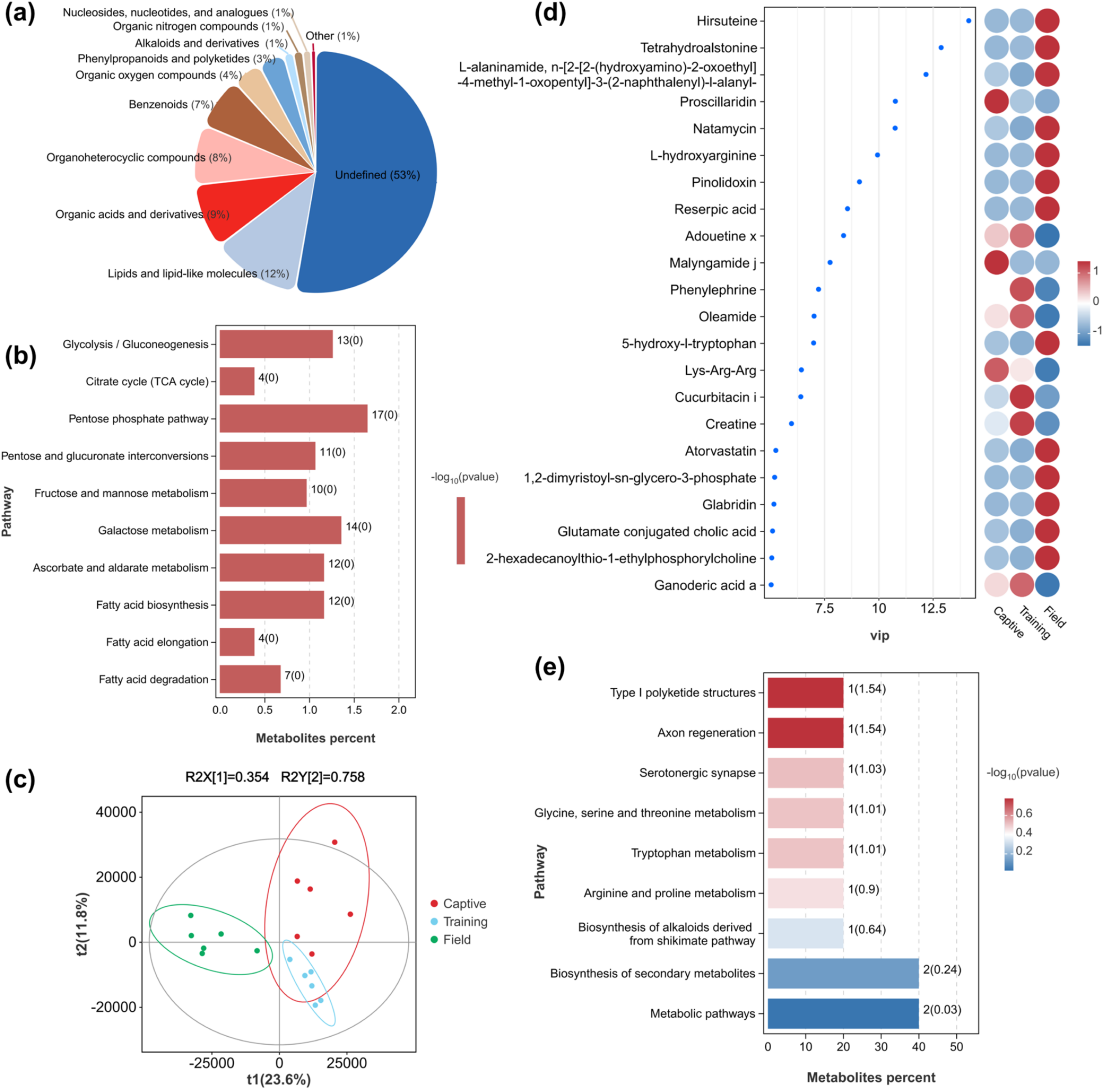
(a) Pie chart of top 10 metabolite attribution information statistics; (b) metabolite KEGG enrichment analysis; (c) PLS‐DA score plot for discriminant analysis; (d) VIP plot showing differential metabolites (VIP value > 5, *p* < 0.05); (e) KEGG enrichment analysis of differential metabolites.

The trend of metabolites during the reintroduction was first assessed using unsupervised principal component analysis (PCA) to determine the stability of the instrument during metabolite data acquisition (Figure S3a). The high clustering of QC samples indicated high system stability and data quality, suggesting that the data are reliable for further analysis. Metabolites from both positive ion (POS) and negative ion (NEG) modes were merged for analysis, and a total of 5605 metabolites were identified. All identified metabolites were classified and statistically analyzed based on the chemical taxonomy affiliation information from the HMDB database. The proportion of the Top 10 metabolites is shown in Figure 4a. Excluding the unclassified, the highest number of metabolites were found in lipids and lipid‐like molecules, organic acids and derivatives, and organoheterocyclic compounds, accounting for 12%, 9%, and 8% respectively. Metabolites were annotated through the KEGG database, and the Top 10 metabolic pathways were displayed through an enrichment bar chart (Figure 4b). It was found that metabolites were primarily annotated and enriched in carbohydrate metabolism and lipid metabolism pathways, with significant enrichment in pathways such as glycolysis/gluconeogenesis and the citrate cycle (TCA cycle), which may play an important role in maintaining the physiological and ecological mechanisms of the Chinese alligator.

Discriminant analysis was performed using the PLS‐DA score plot (Figure 4c), revealing that the reintroduction of the Chinese alligator significantly altered the composition of metabolites in their feces. The permutation test plot (Figure S3b) confirmed the reliability of the PLS‐DA model's predictive results and ruled out overfitting. The criteria for screening differentially expressed metabolites (DEMs) were VIP > 1.00 and *p* < 0.05, leading to the identification of 216 DEMs. The distribution characteristics of the differential metabolites were evident in the metabolite chemical classification statistics plot (Figure S3c), with the majority of metabolites falling into the categories of lipids and lipid‐like molecules, as well as organic acids and their derivatives.

The relative expression characteristics of differential metabolites between comparison groups were displayed using a VIP plot (VIP values > 5, *p* < 0.05) (Figure 4d), revealing that Hirsuteine, Tetrahydroalstonine, and L‐alaninamide (n‐[2‐[2‐(hydroxyamino)‐2‐oxoethyl]‐4‐methyl‐1‐oxopentyl]‐3‐(2‐naphthalenyl)‐l‐alanyl‐), among others, showed significantly increased expression in the field population of the Chinese alligator. Many of these compounds are important in the exploration of drugs and intermediate structures for anti‐inflammatory and antioxidant, antibacterial and antiviral, and antitumor cell research at the pathological level. Proscillaridin, Malyngamide j, and Lys‐Arg‐Arg, on the other hand, were significantly downregulated with the process of reintroduction. KEGG enrichment analysis of differential metabolites (Figure 4e) showed the metabolic pathway enrichment characteristics of the Chinese alligator during the reintroduction process, finding that differential metabolites were mainly enriched in Type I polyketide structures and Axon regeneration pathways. These differences, trends, and pathway enrichments of metabolites reflect the physiological response mechanisms of the Chinese alligator to environmental changes.

### 3.5 Association Analysis of the Microbiome and the Metabolome

**FIGURE 5:**
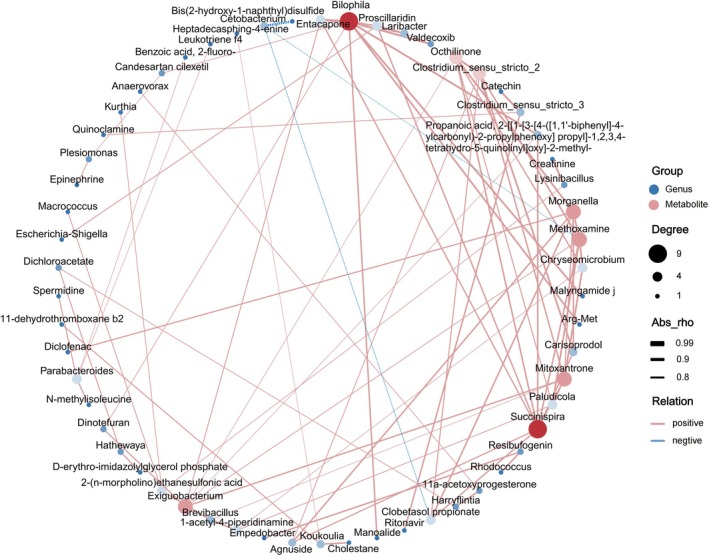
Network plot of differential species and differential metabolite correlation weight (Cor > 0.8, *p* < 0.01).

Species and differential metabolites at the genus level, identified through the Kruskal–Wallis test (*p* < 0.05) and with VIP values > 1 and *p* < 0.05, were subjected to inter‐group correlation analysis based on Pearson's method. Strongly correlated relationships were visualized using a weighted network graph (Correlation > 0.8, *p* < 0.01) (Figure 5).

The results showed that *Succinispira* was significantly positively correlated with differential metabolites such as Proscillaridin, Octhilinone, and Methoxamine. Similarly, *Bilophila* was significantly positively correlated with differential metabolites like Valdecoxib, Proscillaridin, and Propanoic acid. *Succinispira* and *Bilophila* were significantly enriched in the intestines of captive Chinese alligators and significantly downregulated as the process of reintroduction progressed. Additionally, *Cetobacterium*, enriched in the field population, showed significant negative correlations with three differential metabolites: Methoxamine, Clobetasol propionate, and Bis(2‐hydroxy‐1‐naphthyl)disulfide. The correlation and trends of these microbial communities and metabolites may have a significant impact on the synergistic adaptation of the Chinese alligator in both captive and wild environments.

## 4 Discussion

In the ongoing biodiversity crisis, captive conservation and breeding programs have become an important refuge for species survival and have accumulated valuable genetic resources, which are crucial for the future reintroduction of species into the wild (Dallas and Warne 2023). During the process of reintroducing captive populations of protected animals to the wild, the significant disparities between captive and natural environments exert multiple selective pressures on the maintenance and release of these populations. Consequently, this affects the species phenotypic plasticity and genetic adaptability—a process theoretically generalizable across vertebrates yet modulated by taxon‐specific traits (Schulte‐Hostedde and Mastromonaco 2015). In reptiles such as the Chinese alligator, these adaptive processes are further mediated by dynamic host‐microbiome interactions during environmental transition, as demonstrated by our multi‐omics analyses. The reintroduction to the wild is primarily characterized by changes in lifestyle and food conditions, and such transformations play a crucial role in the differentiation of microbial communities (Tang et al. 2020). Integrating 16S rRNA, metagenomic, and metabolomic analyses, this study characterizes the gut microbiome divergence in Chinese alligator populations during various reintroduction phases, identifying diet‐ and habitat‐driven shifts in microbial taxonomy, community assembly, and metabolic potential. These shifts collectively map the ecological restructuring of symbionts during reintroduction.

### 4.1 Adaptive Remodeling of the Microbial Community Structure

The analysis of gut microbiota diversity showed that lifestyle changes during the reintroduction process of the Chinese alligator resulted in a significant increase in the relative abundance of the Fusobacteria within its gut. This increase plays a dominant role in driving changes to the overall ecological niche structure of the Chinese alligator's gut microbiota. The Fusobacteria occupies an ecological niche in the alligator's gut that imparts a distinctive characteristic to its gut microbiome. This uniqueness is likely associated with the host's genetics, dietary habits, niche occupation, and adaptability, playing a vital role in the alligator's health and nutrient absorption (Keenan et al. 2013). Analysis of the Firmicutes‐to‐Bacteroidetes (F/B) ratio indicated a significant increase in this ratio among field populations. The F/B ratio can be employed as a straightforward evaluative indicator, with previous research implicating it in more efficient energy absorption and fat accumulation. (Magne et al. 2020). The F/B ratio in the field population of the Chinese alligator has significantly increased compared to both captive and domesticated populations, which may be correlated with the relatively challenging acquisition of food in the wild. As a result, the gut microbiota may adapt to help the host better utilize scarce resources and optimize energy acquisition and utilization.

The relative abundance of *Cetobacterium*, belonging to the phylum Fusobacteria, has significantly increased and taken a dominant ecological niche process in the gut microbiota of both domesticated and field populations. *Cetobacterium* is known to dominate the gut microbiota of many freshwater fish species, where it can activate the parasympathetic nervous system through its metabolic product acetate, promoting the host's expression of insulin and enhancing the ability to utilize carbohydrates (Wang et al. 2021). It also acts as a gut microbiota regulator by synthesizing vitamin B12, which strengthens the interactions within the gut microbiota and improves the host's resistance to pathogen infections (Qi et al. 2023). At the same time as the significant increase in the relative abundance of *Cetobacterium*, there has been an observed trend in the relative abundance changes of some typical conditional pathogenic bacteria. For example, the relative abundance of typical pathogens such as *Edwardsiella*, *Escherichia–Shigella*, and *Comamonas* has significantly decreased (Aggarwal et al. 2019; Liu et al. 2020; Ryan et al. 2022). These trends in species changes indicate the characteristic of synergistic adaptation between the gut microbiota and the host during the process of releasing the Chinese alligator into the reintroduction.

Gut type is a population stratification that displays individual variation based on the composition of the gut microbiota, revealing the presence of several ecological types in humans and various animal species, which may be associated with different capacities and susceptibilities of their hosts (Larzul et al. 2024). Analysis has revealed that the gut type of the Chinese alligator undergoes differentiation during the process of reintroduction, with the type originally dominated by *Acinetobacter* gradually being replaced by *Cetobacterium*. Neutral Community Models (NCM) have shown that the assembly mechanism of the gut microbial community in the Chinese alligator is predominantly driven by neutral (random) processes. However, the transition to reintroduction—that is, changes in environmental and dietary conditions—causes the gut microbial community to trend towards deterministic processes. This indicates that during the process of releasing the Chinese alligator into the wild, environmental selection and interactions between species begin to play a more significant role in the construction of the gut microbial community. Changes in gut type and community assembly mechanisms further validate the adaptive evolution of the gut microbiota during the reintroduction process of the Chinese alligator.

### 4.2 Adaptation and Underlying Mechanisms of Gut Microbial Community Function

Functional annotation analysis of the gut microbiota was used to characterize the adaptability and underlying mechanisms of the gut microbial community function in the Chinese alligator during the process of reintroduction. First, annotation through the KEGG database revealed that the expression of the Two‐component system (TCS) gene pathway in the Pathway category decreased as the reintroduction process progressed. The Two‐component system plays a significant role in adaptive and virulence regulation in bacteria. It enables bacteria to respond to environmental changes through sensing and to regulate their adaptability and virulence, allowing them to survive and cause disease in changing environments (Xie et al. 2022).

Analysis using the Kruskal–Wallis test at the Pathway classification level revealed that the expression of the Proteoglycans in the cancer pathway was significantly upregulated as the process of reintroduction progressed. Proteoglycans are key molecular effectors on the cell surface and in the pericellular microenvironment, and due to their polyvalent nature and their ability to interact with ligands and receptors that regulate tumor growth and angiogenesis, they play various roles in cancer and angiogenesis (Iozzo and Sanderson 2011). Meanwhile, proteoglycans can be used as potential targets for cancer therapy, playing important roles in the regulation of immune response, the activation of matrix‐degrading enzymes, and the binding of signaling molecules such as chemokines and cytokines in immune cells (Savage et al. 2024). The Flagellar assembly and the biosynthesis of various other secondary metabolites pathways were significantly downregulated as the reintroduction process progressed. The flagellar assembly process involves the synergistic work of numerous proteins, including the formation of the basal body, hook structure, and filament assembly. The motility driven by flagella is crucial for the pathogenicity of many gut pathogens, such as *Salmonella Typhimurium* (Akahoshi and Bevins 2022). Further characterization has revealed that the reintroduction process has led to a reduction in the pathogenic risk of the gut microbiota in Chinese alligators.

Annotation from the CAZy database revealed that the highest number of genes enriched were in glycoside hydrolases (GH) families associated with ‘carbohydrate metabolic processes’. Notably, GH13 and GH23 emerged as dominant CAZymes within the GH family. The GH13 family, a principal α‐amylase family, exhibits increased activity that correlates with higher levels of short‐chain fatty acids, such as butyrate, in the colon. This increase significantly affects the host's metabolic health, particularly in the regulation of fat deposition and energy balance (Stam et al. 2006; Ma et al. 2024). Enzymes of the GH23 family, which include lysozymes and endo‐β‐N‐acetylglucosaminidases, play a variety of significant physiological roles in animal bodies, particularly in the immune defense and pathogen defense mechanisms, where they exert critical functions (Wohlkönig et al. 2010; Chen et al. 2023; Martinez‐Bond et al. 2022).

Enzymes from the GH13 and GH23 families may play roles in various biological processes within the body of the Chinese alligator, such as energy metabolism and host‐pathogen interactions. Through further analysis using the Kruskal–Wallis test at the Level‐B classification level, it was found that the GT19 are significantly upregulated as part of the reintroduction process. Enzymes from the GH19 family, such as chitinases, may positively contribute to resistance against fungal infections. In nature, chitinases can break down chitin and convert it into valuable products, such as chitooligosaccharides, which possess anti‐inflammatory and antibacterial properties (Sha et al. 2022). With the reintroduction of the Chinese alligator, the expression levels of CBM6, GH121, and GT101 significantly decrease. The enzymes from the CBM6, GH121, and GT101 families may play important roles in gut infections by enhancing the adhesion capability of pathogens, altering the polysaccharide structure on the bacterial surface, promoting the colonization of pathogens, and participating in the immune evasion mechanisms of pathogens (Abbott et al. 2009; Saito et al. 2020).

Furthermore, genes were annotated to the Comprehensive Antibiotic Resistance Database (CARD) for annotation analysis, revealing that the number of ARO_name genes within the gut is downregulated, with the expression level of antibiotic resistance genes (ARGs) being highest for macB. The macB gene codes for an ABC transporter protein, which may increase bacterial resistance by expelling certain antibiotics from bacterial cells via efflux pump systems such as MacAB‐TolC, for instance, macrolide drugs (Greene et al. 2018). Differential analysis revealed that the antibiotic resistance genes RbpA, arr‐3, vanH from the vanF cluster, and tet(D) are significantly downregulated with the progression of the reintroduction process. These genes are primarily associated with rifamycins, aminoglycosides, vancomycin, and tetracyclines. The use of drugs represented by macrolide antibiotics may lead to the enrichment of ARGs (Antibiotic Resistance Genes). These genes can be transmitted through the food chain and could ultimately impact the health of the Chinese alligator.

### 4.3 Validation Characterization of Metabolites

The metabolites detected in the feces of the Chinese alligator are dominated by the group of lipids and lipid‐like molecules. Lipids are important cellular components for maintaining internal balance in the presence of environmental stressors and are crucial for the survival and reproduction of animals under environmental stress conditions (Lee et al. 2018).

Through KEGG enrichment annotation analysis, it was discovered that the metabolites are mainly enriched in the glycolysis/gluconeogenesis pathway, which is a key metabolic pathway for carbohydrate metabolism. Glycolysis/gluconeogenesis is a vital metabolic pathway in animals, playing a significant role in energy supply and adaptation to environmental changes. Glycolysis is a critical pathway for converting glucose into energy, especially under anaerobic conditions such as during intense exercise, where animals rely on ATP produced by glycolysis to meet their energy requirements (Kierans and Taylor 2021). Gluconeogenesis is crucial during prolonged fasting or starvation states as it helps maintain blood glucose levels, ensuring that tissues with a high demand for glucose, such as the brain and red blood cells, can function normally (Zhao et al. 2023). The significant enrichment of the glycolysis and gluconeogenesis pathways may interact with the survival strategies of the Chinese alligator, supporting their maintenance of homeostasis during extended periods of diving, hunting, and relative scarcity of food, thus ensuring their survival capabilities in extreme environments.

Through differential metabolite analysis, it was found that the content of metabolites represented by Hirsuteine and Tetrahydroalstonine is significantly upregulated in field populations. Hirsuteine is an alkaloid with multiple pharmacological effects, including neuroprotection, anticonvulsant, and blood pressure‐lowering properties. In model animals, it has shown potential therapeutic effects on central nervous system diseases and exerts a cellular protective role through antioxidant and anti‐apoptotic mechanisms (Qi et al. 2022; Ndagijimana et al. 2013). Tetrahydroalstonine exhibits good protective effects against neuronal damage induced by OGD/R through auTophagy regulation, demonstrating its potential in neuroprotection (Liao et al. 2023). These two metabolites are associated with neuroendocrine immune regulation. They exert neuroprotective and immunomodulatory effects by influencing mechanisms such as neurotransmission in the nervous system, neurotransmitter levels, and auTophagy functions. The upregulation of these alkaloids has a positive impact on the cognitive functions and behavioral adaptability of the Chinese alligator when exposed to increased environmental stressors in the wild, such as food acquisition, exploratory behavior, and adaptation to climate change. Proscillaridin and Malyngamide J both significantly decrease with the progression of the reintroduction process. Proscillaridin and Malyngamide J have both shown significant biological activity in animal models. Proscillaridin primarily functions by enhancing myocardial contractility and inhibiting the growth of cancer cells, but this mechanism can also lead to cardiac toxicity, such as arrhythmias (Denicolaï et al. 2014; Luo et al. 2021). Malyngamide J primarily exhibits anti‐inflammatory and immunomodulatory effects, but in some studies, it has shown toxicity to shrimp and goldfish, indicating that it may be toxic to aquatic organisms at high concentrations (Villa et al. 2010; Jiang et al. 2018). The significant downregulation of this compound may be associated with changes in environmental conditions during the process of reintroduction.

The KEGG metabolic pathway enrichment analysis of differential metabolites revealed that these metabolites are mainly enriched in the Type I polyketide structures (PKS) and Axon regeneration pathways. The role of PKS in signaling pathways is primarily reflected in its biosynthetic mechanisms and regulatory networks, making it a class of major natural products used as antiviral, antibiotic, antifungal, antiparasitic, immunosuppressive, and antitumor drugs (Yan et al. 2024). Axon regeneration involves multiple signaling pathways that play a crucial role in the regeneration and repair processes of neurons. Indole‐3‐propionic acid (IPA), a derivative of the microbiome, can promote the regeneration and functional recovery of sensory axons through immunomodulatory mechanisms (Serger et al. 2022; Hisamoto and Matsumoto 2017)。The significant enrichment of these metabolic pathways indicates that when the Chinese alligator transitions from a captive environment to the wild, there is an increased demand for nutrients, enhanced resistance to environmental stress, and improved neuroendocrine immune regulatory capacity. This reflects the adaptive changes in physiological regulation of the Chinese alligator in response to the wild environment.

### 4.4 Joint Analysis Characterizes the Adaptation Process of the Chinese Alligator's Gut Microbiome Mediating Host Metabolism

*Succinispira* can influence the host's metabolism and immune system in various ways through the production of succinic acid, including regulating blood glucose levels, promoting gut gluconeogenesis, activating gut immune responses, and participating in the maintenance of the host's energy homeostasis (De Vadder et al. 2016; Nadjsombati et al. 2018). *Succinispira* shows a significant positive correlation with various metabolites that exhibit significant anti‐tumor activity, such as Proscillaridin, Octhilinone, and Mitoxantrone (El‐Sayed et al. 2024; Evison et al. 2016). *Bilophila* is a conditional pathogen, and an increase in its abundance is associated with negative effects on gut inflammation. Its synergistic effect with a high‐fat diet may promote higher inflammatory responses, gut barrier dysfunction, and abnormalities in bile acid metabolism, leading to abnormal glucose metabolism and hepatic steatosis (Olson et al. 2021). *Succinispira* and *Bilophila* are significantly enriched in the gut of captive Chinese alligators, but both show a significant decrease with the progression of the reintroduction process (Park et al. 2022; Li, Lin, et al. 2022). In addition, the field population has a significant enrichment of *Cetobacterium*, which is significantly negatively correlated with three differential metabolites: Methoxamine, Clobetasol propionate, and IPA‐3, which act as adrenergic receptor agonists, anti‐inflammatory antibiotics, and tumor kinase inhibitors, respectively (Li, Li, et al. 2022; Kohutova et al. 2023; Wright et al. 2020).

The research reveals that the expression changes and trends of pathways, genes, and metabolites mediated by the microbiome reflect the response strategies of the Chinese alligator gut microbiome to different environmental scales. In the crocodile population, the composition of the gut microbiome is related to the lifespan and cellular aging, genome and epigenome of crocodiles, as well as their resistance to infectious diseases and cancers. Its highly adaptive and efficient immune system has co‐evolved closely with the resident microbiota (Keenan and Elsey 2015; Siddiqui et al. 2021). Our study reveals adaptive interactions between crocodilian gut microbiomes and hosts, providing a framework to identify healthy host‐microbe symbiosis in other endangered species. Microbiome dynamics under environmental shifts serve as biomarkers for health assessment and adaptability prediction, while insights into host‐microbe co‐evolution can enhance captive breeding programs. Multi‐omics integration enables targeted conservation strategies by linking microbial functions to species resilience.

## 5 Conclusion

This study utilized multi‐omics techniques to investigate gut microbiome and metabolic shifts in Chinese alligators across captive, training, and field populations. Key findings include: (1) Reduced microbial diversity and increased dominance of Fusobacteriota (e.g., *Cetobacterium*) during reintroduction, with community assembly shifting from neutral to deterministic processes. (2) Functional depletion of pathogenic genes (e.g., antibiotic resistance) and enrichment of carbohydrate metabolism pathways (e.g., glycolysis), potentially reflecting dietary adaptation to wild prey. (3) Differential metabolite profiles between populations—neuroprotective alkaloids (e.g., hirsutine) in Field Chinese alligators versus immunomodulatory compounds in captive individuals—suggesting microbiome‐mediated metabolic adjustments to environmental transitions. These associations may inform the development of microbial biomarkers (e.g., *Cetobacterium* abundance) for monitoring reintroduction preparedness. However, causal links between microbiome dynamics and host adaptation (e.g., stress resistance, survival) remain speculative without physiological validation. Future work integrating host phenotyping and microbial activity measurements will clarify the conservation relevance of these multi‐omics signatures.

## Author Contributions

**Chong Wang:** conceptualization (lead), data curation (lead), formal analysis (lead), investigation (lead), methodology (lead), software (lead), visualization (lead), writing – original draft (lead). **Changcheng Li:** data curation (equal), funding acquisition (equal), methodology (equal), software (equal). **Fuyong You:** investigation (equal), software (equal), visualization (equal). **Yongkang Zhou:** investigation (equal), project administration (equal), resources (equal), supervision (equal). **Genjun Tu:** investigation (equal), resources (equal). **Ruoya Liu:** investigation (equal), resources (equal). **Pingsi Yi:** investigation (equal), resources (equal). **Xiaobing Wu:** conceptualization (equal), funding acquisition (equal), investigation (equal), project administration (equal), resources (equal), supervision (equal), writing – review and editing (equal). **Haitao Nie:** conceptualization (equal), funding acquisition (equal), investigation (equal), project administration (equal), resources (equal), supervision (equal), writing – review and editing (equal).

## Conflicts of Interest

The authors declare no conflicts of interest.

## Supporting information


**Figure S1.** (a) Sob Sparse curve; (b) Upset plot showing the unique and shared OUT number characteristics; (c) Anosim test; (d) Kruskal–Walli testat phylum level; (e) Tukey HSD ratio analysis of Firmicutes to Bacteroides; (f) Kruskal–Walli test at phylum level; (g) Calinski‐Harabasz (CH) index.

**Figure S2.** (a) Upset Figure shows the number characteristics of Pathway pathways between the three groups; (b) The Kruskal–Walli test of the Pathway level; (c) Upset diagram showing the number characteristics of Level‐B pathways between the three groups in the CAZy database; (d) The Kruskal–Walli test for the Level‐B level; (e) Upset diagram showing the number of ARO _ name gene in CARD database; (f) Circos Figure showing the Kruskal–Walli test of the functional distribution pattern of antibiotic resistance genes (ARGs) of TOP10; (g) The Kruskal–Walli test for the ARO _ name level.

**Figure S3.** (a) PCA shows the aggregation of QC samples; (b) PLS‐DA model displacement test map; (c) chemical classification statistics map of differential metabolites.

**Table S1.** The comparative distribution of samples pre‐ and post‐screening with MicroPITA analyses.
**Table S2.** Assembly statistics for each sample.
**Table S3.** Database annotation statistics.

## Data Availability

The raw sequence data reported in this paper have been deposited in the Genome Sequence Archive in National Genomics Data Center, China National Center for Bioinformation/Beijing Institute of Genomics, Chinese Academy of Sciences (GSA: CRA020378 and 020375) that are publicly accessible at https://ngdc.cncb.ac.cn/gsa.
