# Supplementary figures and images for: Multi‐Omics Analysis of Gut Microbiome and Host Metabolism in Different Populations of Chinese Alligators (alligator sinensis) During Various Reintroduction Phases
Source: Ecol Evol. 2025 Apr 9;15(4):e71221. doi: 10.1002/ece3.71221 (PMC11981878; doi:10.1002/ece3.71221)

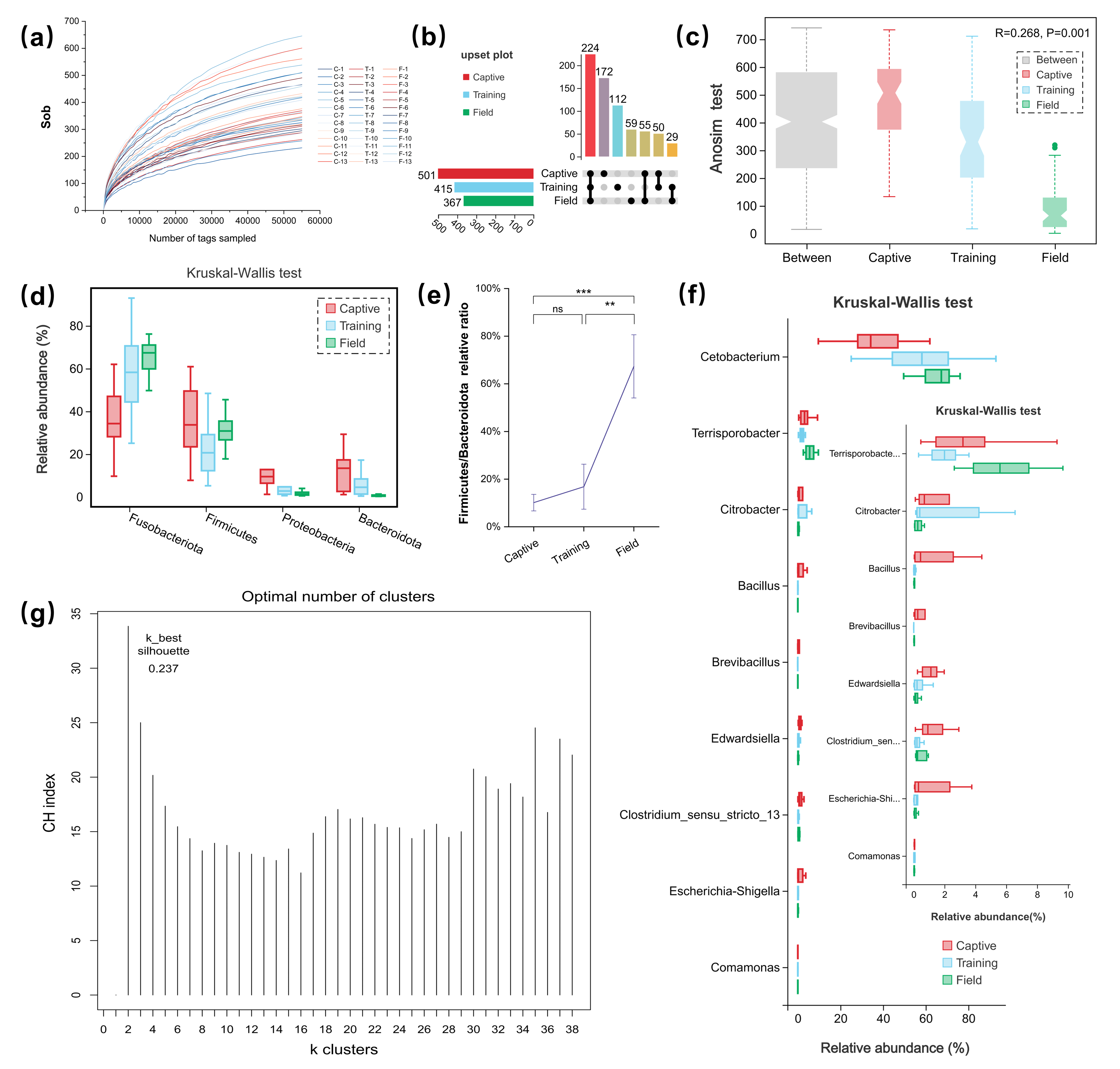

Supplement: Supplementary file 1 — Figure S1. (a) Sob Sparse curve; (b) Upset plot showing the unique and shared OUT number characteristics; (c) Anosim test; (d) Kruskal–Walli testat phylum level; (e) Tukey HSD ratio analysis of Firmicutes to Bacteroides; (f) Kruskal–Walli test at phylum level; (g) Calinski‐Harabasz (CH) index. [file ECE3-15-e71221-s003.tif]

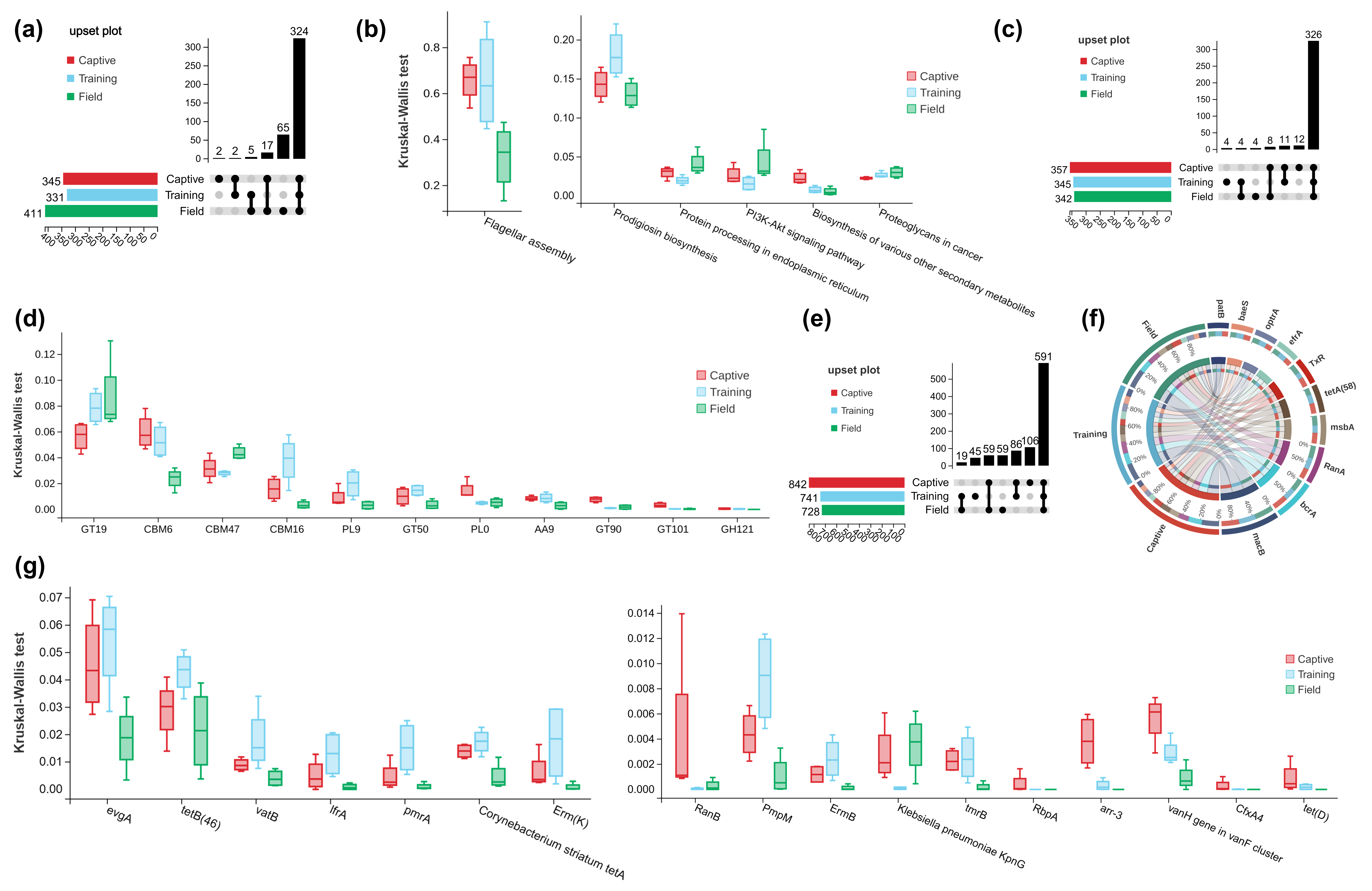

Supplement: Supplementary file 2 — Figure S2. (a) Upset Figure shows the number characteristics of Pathway pathways between the three groups; (b) The Kruskal–Walli test of the Pathway level; (c) Upset diagram showing the number characteristics of Level‐B pathways between the three groups in the CAZy database; (d) The Kruskal–Walli test for the Level‐B level; (e) Upset diagram showing the number of ARO _ name gene in CARD database; (f) Circos Figure showing the Kruskal–Walli test of the functional distribution pattern of antibiotic resistance genes (ARGs) of TOP10; (g) The Kruskal–Walli test for the ARO _ name level. [file ECE3-15-e71221-s001.tif]

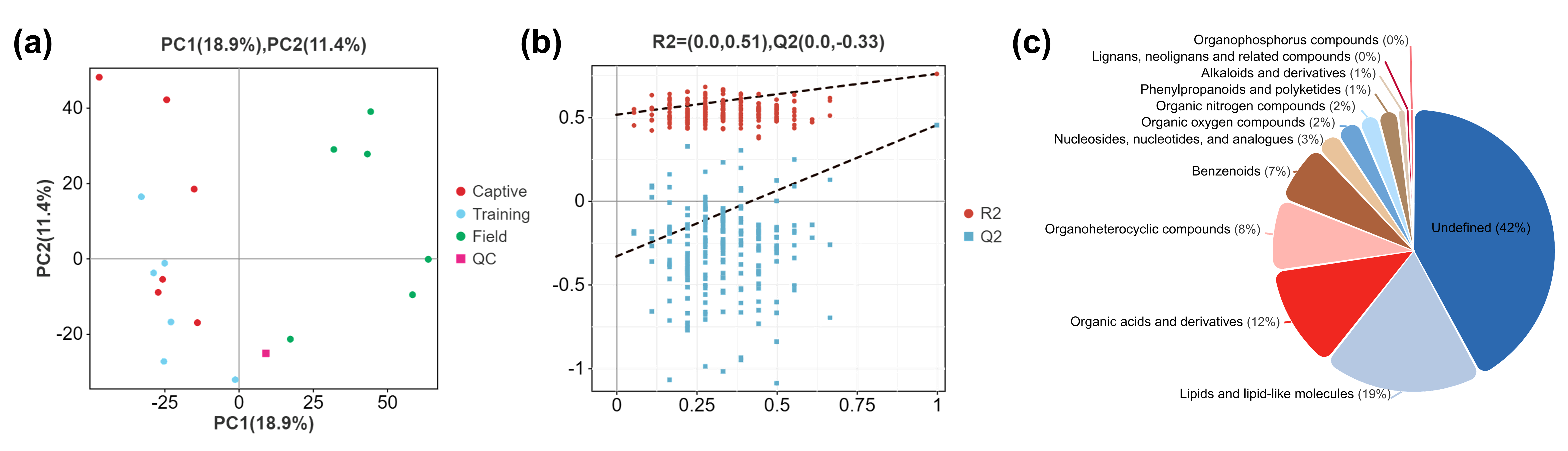

Supplement: Supplementary file 3 — Figure S3. (a) PCA shows the aggregation of QC samples; (b) PLS‐DA model displacement test map; (c) chemical classification statistics map of differential metabolites. [file ECE3-15-e71221-s004.tif]
