# Supplementary material for: Multi‐Omics Analysis of Gut Microbiome and Host Metabolism in Different Populations of Chinese Alligators (alligator sinensis) During Various Reintroduction Phases
Source: Ecol Evol. 2025 Apr 9;15(4):e71221. doi: 10.1002/ece3.71221 (PMC11981878; doi:10.1002/ece3.71221)
Supplement: Supplementary file 4 — Table S1. The comparative distribution of samples pre‐ and post‐screening with MicroPITA analyses. Table S2. Assembly statistics for each sample. Table S3. Database annotation statistics. [file ECE3-15-e71221-s002.docx]

**Supplementary Material**

**Table S1 The comparative distribution of samples pre- and post-screening with MicroPITA analyses.**

| **Various Reintroduction Phases** | **Initial sample** | **Metagenomics** | **Metabonomics** | **Remaining samples (no metagenomics / metabolomics analysis performed) *** |
| --- | --- | --- | --- | --- |
| **Captive** | 13 | 4 (30.8%) | 6 (46.2%) | 9 (69.2%) /7 (53.8%) |
|  | C1-C13 | C_1, C_3, C_4, C_12 | C_1, C_3, C_4, C_12, C_6, C_11 | C2, C5-C11, C13/C2, C5, C7, C8, C9, C10, C13 |
| **Training** | 13 | 4 (30.8%) | 6 (46.2%) | 9 (69.2%) /7 (53.8%) |
|  | T1-T13 | T_2, T_5, T_9, T_11 | T_2, T_5, T_9, T_11, T_8, T_13 | T1, T3, T4, T6-8, T10, T12-13/T1, T3, T4, T6, T7, T10, T12 |
| **Field** | 13 | 4 (30.8%) | 6 (46.2%) | 9 (69.2%) /7 (53.8%) |
|  | F1-F13 | F_6, F_7, F_9, F_10 | F_6, F_7, F_9, F_10, F_8, F_13 | F1-5, F8, F11-13/F_1, F_2, F_3, F_4, F_5, F_11, F_12 |

*The sample distribution before and after screening was strictly balanced between groups, and the remaining samples were defined as samples that passed QC but were not selected for downstream analysis (metagenome residual: 39-12=27; metabolome residual: 39-18=21). Complete data was not displayed in the text due to space constraints.

**Table S2 Assembly statistics for each sample**

| **Sample** | **Contigs Num** | **Total length** | **Average length** | **Max length** | **Min length** | **N50** | **N90** |
| --- | --- | --- | --- | --- | --- | --- | --- |
| C_1 | 118573 | 182771410 | 1541.43 | 462750 | 500 | 2105 | 632 |
| C_3 | 98513 | 143137027 | 1452.98 | 670045 | 500 | 1875 | 621 |
| C_4 | 189578 | 298838383 | 1576.33 | 228294 | 500 | 2267 | 637 |
| C_12 | 144486 | 216833577 | 1500.72 | 801304 | 500 | 1998 | 631 |
| T_2 | 125777 | 198324607 | 1576.80 | 361976 | 500 | 2262 | 637 |
| T-5 | 127091 | 207143690 | 1629.88 | 517039 | 500 | 2482 | 638 |
| T_9 | 76037 | 124791707 | 1641.20 | 967352 | 500 | 2689 | 618 |
| T_11 | 102318 | 173417462 | 1694.89 | 262868 | 500 | 2559 | 659 |
| F_6 | 52572 | 86486138 | 1645.10 | 237045 | 500 | 2283 | 658 |
| F_7 | 56933 | 102908488 | 1807.54 | 324337 | 500 | 3519 | 642 |
| F_9 | 84517 | 127533714 | 1508.97 | 317518 | 500 | 2000 | 635 |
| F_10 | 159786 | 270641589 | 1693.78 | 626177 | 500 | 2513 | 667 |

**Table S3 database annotation statistics**

| **Database** | **GeneCount** | **GenePercent(%)** |
| --- | --- | --- |
| KEGG | 925504 | 86.12% |
| CAZy | 178674 | 16.63% |
| CARD | 47453 | 4.42% |
